# KSHV infection skews macrophage polarisation towards M2-like/TAM and activates Ire1 α-XBP1 axis up-regulating pro-tumorigenic cytokine release and PD-L1 expression

**DOI:** 10.1038/s41416-020-0872-0

**Published:** 2020-05-18

**Authors:** Maria Saveria Gilardini Montani, Luca Falcinelli, Roberta Santarelli, Marisa Granato, Maria Anele Romeo, Nives Cecere, Roberta Gonnella, Gabriella D’Orazi, Alberto Faggioni, Mara Cirone

**Affiliations:** 1 Department of Experimental Medicine, Sapienza University of Rome, laboratory affiliated to Istituto Pasteur Italia-Fondazione Cenci Bolognetti, Rome, Italy; 2 Department of Research, Advanced Diagnostics, and Technological Innovation, Regina Elena National Cancer Institute, Rome, Italy; 3 Department of Medical, Oral and Biotechnological Sciences, University “G. D’Annunzio”, 66100 Chieti, Italy

**Keywords:** Stress signalling, Cancer microenvironment

## Abstract

**Background:**

Kaposi’s Sarcoma Herpesvirus (KSHV) is a gammaherpesvirus strongly linked to human cancer. The virus is also able to induce immune suppression, effect that contributes to onset/progression of the viral-associated malignancies. As KSHV may infect macrophages and these cells abundantly infiltrate Kaposi’s sarcoma lesions, in this study we investigated whether KSHV-infection could affect macrophage polarisation to promote tumorigenesis.

**Methods:**

FACS analysis was used to detect macrophage markers and PD-L1 expression. KSHV infection and the molecular pathways activated were investigated by western blot analysis and by qRT-PCR while cytokine release was assessed by Multi-analyte Kit.

**Results:**

We found that KSHV infection reduced macrophage survival and skewed their polarisation towards M2 like/TAM cells, based on the expression of CD163, on the activation of STAT3 and STAT6 pathways and the release of pro-tumorigenic cytokines such as IL-10, VEGF, IL-6 and IL-8. We also found that KSHV triggered Ire1 α-XBP1 axis activation in infected macrophages to increase the release of pro-tumorigenic cytokines and to up-regulate PD-L1 surface expression.

**Conclusions:**

The findings that KSHV infection of macrophages skews their polarisation towards M2/TAM and that activate Ire1 α-XBP1 to increase the release of pro-tumorigenic cytokines and the expression of PD-L1, suggest that manipulation of UPR could be exploited to prevent or improve the treatment of KSHV-associated malignancies.

## Background

Kaposi’s sarcoma herpes virus (KSHV) is an oncogenic virus belonging to gammaherpesvirus family associated with several human malignancies such as Kaposi’s sarcoma, primary effusion lymphoma (PEL) and Castleman’s disease.^1^ KSHV encodes for many proteins homologue to human proteins that favour oncogenesis, counteract apoptosis and subvert immune response such as viral G-protein-coupled receptors (GPCRs), viral Cellular FLICE-inhibitory protein (FLIP) and viral interleukin 6 (IL-6). Among the strategies through which KSHV induces immune suppression there is the impairment of dendritic cell (DC)^2,3^ essential for anti-tumour immune response^4^ or macrophage formation,^5^ to dysregulate the release of cytokines by virus-infected DCs^6^ or endothelial cells, promoting macrophage polarisation into M2-like/tumour associated macrophage (TAM).^7^ Macrophages are plastic cells that can undergo polarisation by shifting between pro-inflammatory M1 and anti-inflammatory M2 functional phenotypes.^8^ TAM are a subgroup of M2 that among other cytokines may produce VEGF that, besides reducing immune response promote angiogenesis and tumour survival/progression^9^ or may release molecules that contribute to extracellular matrix degradation.^10^ Even if macrophages can be target of KSHV infection^11^ the impact of KSHV infection on their polarisation has not been investigated yet. In this study we investigated whether KSHV infection could skew macrophage polarisation towards M2-like/TAM and co-opt these cells to promote tumorigenesis, also considering that the virus has been reported to induce the phosphorylation of Signal Transducer and Activator of Transcription (STAT) 3^6^ and STAT6^12^ in other cell types, pathways activated in M2-like/TAM macrophages.^13–15^ STAT3 and STAT6 phosphorylation may activate the unfolded protein response (UPR), in particular the Inositol-requiring enzyme 1 (Ire1) α endoribonuclease, in bone marrow-derived macrophages.^16^ Ire1α, the most conserved signalling branch of UPR, is an endoplasmic reticulum (ER) kinase that, among other functions, can induce the splicing of X-box binding protein 1 (XBP1), generating the transcription factor XBP1s. Ire1α, together with the other ER kinase, such as protein kinase R (PKR)-like endoplasmic reticulum kinase (PERK) and activating transcription factor 6 (ATF6), orchestrate the UPR in response to ER stress. Besides by inducing STAT3 and STAT6 phosphorylation, KSHV could activate UPR by triggering ER stress, as the cellular translation machinery is hijacked by viruses, particularly during the replicative phase of their life cycle, to produce the large amount of proteins required for viral replication.^17^ Moreover, in the course of microbial infection, UPR could be, independently of ER stress, activated by the pattern recognition receptor (PRR) signalling.^18^ It is known that UPR, depending on the intensity and duration of ER stress may up-regulate pro-survival molecules such as binding immunoglobulin protein (BIP), promoting the adaption to cell stress, or increase the expression of pro-death molecules such as C/EBP homologous protein (CHOP).^19^ However, UPR may affect several cellular processes, i.e. it may affect immune response even if the consequences of the activation of the different UPR arms on immune cell function has just begun to be investigated. For example it has been observed that ATF4 and CHOP up-regulation, that mainly occurs downstream of PERK, occurred in myeloid-derived suppressor cells (MDSCs) present in the tumour environment.^20^ Ire1 α-XBP1 axis has been reported to influence the function of dendritic cells^21,22^ or macrophages.^16^ Interestingly UPR, especially the most studied branches PERK and Ire1α, may also influence cytokine production by immune cells.^18,23^ Therefore, in this study we next investigated whether KSHV infection could activate Ire1α and PERK branches of UPR in macrophages and if this effect increases the release of cytokines promoting tumorigenesis. Finally the expression of programmed death-ligand 1 (PD-L1), an immune check point inhibitor whose expression has been reported to be influenced by UPR in tumour cells^24^ and to occur in KSHV-infected monocytes,^25^ was evaluated in KSHV-infected macrophages and correlated with Ire1α and PERK activation. Unveiling the molecular mechanisms through which KSHV dysregulates the immune response could allow specific targeting of molecules promoting KSHV-associated malignancies.

## Methods

### Monocytes isolation, macrophage differentiation and KSHV infection

Monocytes isolated from human peripheral blood mononuclear cells (PBMCs) of healthy donors as previously described^26,27^ were cultured in RPMI 1640 (Euroclone, ECB9006L) containing 10% FCS, 2 mM l-glutamine, 100 U/ml penicillin and 100 mg/ml streptomycin (complete medium) with the addition of 50 ng/ml recombinant human macrophage-colony stimulating factor M-CSF (Peprotech, 300-25) every two days for 6/7 days to differentiate in macrophages. Macrophages were infected with KSHV, obtained as previously described, at a multiplicity of infection (MOI) of 10 genome equivalents/cell for 1 h at 37 °C, or mock-infected and then cultured for additional 24 h in 10% foetal calf serum (FCS) RPMI 1640 medium.^2,3^ In some experiments, macrophages were pre-treated for 1 h with the Ire1α inhibitor 4μ8c (30 μM, Sigma Aldrich, SML0949) or the PERK inhibitor GSK 2606414 (1 μM, Calbiochem, 516535) before infection. Macrophages were left untreated (M0) or polarised towards M1 or M2 macrophages by LPS and IFN gamma (100 ng/ml and 20 ng/ml) or IL-4 (25 ng/ml), respectively, added for 24 h.

HUVECs cells cultured in endothelial cell growth medium (EBM, CC-3121, Lonza) containing EGM SingleQuotes (CC-4133), were KSHV-infected or mock-infected for 2 h at 37 °C and then plated in complete medium supplemented or not with 0.22 μm filtered supernatant of KSHV-infected, or UV-KSHV treated macrophages for 96 h. UV-KSHV inactivation was carried out at 1500 mJ in a cross-linker for 10 min (Spectrolinker XL-1500 UV crosslinker).

### RNA isolation and quantitative real-time PCR analysis

Total RNA was extracted from cells by using TRIzReagent (Invitrogen, Carlsbad, CA, USA) in accordance with manufacturer’s instructions. PCR analyses were carried out using the following specific oligonucleotides:

LANA forw CGGAGCTAAAGAGTCTGGTG- LANA rev GCAGTCTCCAGAGTCTTCTC

ORF50forwCACAAAAATGGCGCAAGATGA- ORF50revTGGTAGAGTTGGGCCTTCAGTT

K8.1forw TAAACGGGACCAGACTAGCAGC- K8.1rev GTTTTCTGCGACCGGTGATACG

ACTforwTCACCCACACTGTGCCATCTACGA-Actrev CAGCGGAACCGCTCATTGCCAAT GG. Transcripts were measured by real-time PCR using the SYBR Green assay (Applied Biosystems, Carlsbad, CA, USA) with a StepOne instrument and 7500 Fast Real-Time PCR System (Applied Biosystems). All primer sets worked under identical quantitative PCR cycling conditions with similar efficiencies to obtain simultaneous amplification in the same run. The 2^−ΔΔC*T*^ method for relative quantitation of gene expression was used to determine mRNA expression levels. *β-actin* gene expression was used as endogenous controls to standardise mRNA expression. All reactions were run in triplicate.

### Cell viability

After 24 h of KSHV infection, a trypan blue (Euroclone) exclusion assay was performed to assess cell viability of uninfected (mock) or KSHV-infected macrophages. Live cells were counted by light microscopy using a Neubauer haemocytometer. The experiments were performed in triplicate and repeated at least three times.

### Immunofluorescence staining and FACS analysis

After 24 h, uninfected, KSHV-infected or UV-KSHV treated macrophages were stained with antibodies against CD86 (Miltenyi Biotec, 130-094-877), CD163 (Santa Cruz Biotechnology, sc20066), PD-L1 (Biolegend, 329706) and isotype control antibody (Miltenyi Biotec, 130-095-897) and analysed by FACS Calibur flow cytometer (BD Transduction Laboratories), using CELLQuest software (BD Biosciences).^28^ Debris and dead cells were excluded from the analysis, gating live cells in a forward versus side scatter (FSC vs SSC) density plot. For each analysis 10.000 events were recorded.

### Western blot analysis

In all, 1 × 10^6^ uninfected or KSHV-infected cells were lysed, subjected to electrophoresis and transferred to nitrocellulose membranes, as previously described.^29^ Membranes were blocked in PBS-0.1% Tween 20 solution containing 3% BSA, probed with specific antibodies and developed using ECL Blotting Substrate (Advansta). The following antibodies were used: mouse monoclonal antibody against Kb-ZIP (Santa Cruz Biotechnology, sc-69797), pSTAT6 (1:100; Santa Cruz Biotechnology Inc., sc-136019), STAT6 (1:100; Santa Cruz Biotechnology Inc., sc-1689), mouse monoclonal anti-STAT3 (1:1000; BD Transduction Laboratories, 610189), mouse monoclonal anti-phospho-STAT3 (p-Tyr705, 1:100; Santa Cruz Biotechnology Inc., sc-8059), pSTAT1 (1:100; Santa Cruz Biotechnology Inc., sc-136229), STAT1 (1:100; Santa Cruz Biotechnology Inc., sc-464), mouse monoclonal anti-Ire1α (1:100; Santa Cruz Biotechnology, sc-390960), XBP1s (NovusBio NBP1-77681SS), ATF4 (R&D system, MAB7218), rabbit polyclonal anti-BIP (1:100; Cell Signaling, 3177), mouse monoclonal anti-CHOP (1:100; Santa Cruz Biotechnology, sc-7351), and anti-β-actin (1:10000; Sigma Aldrich, A5441). Goat anti-mouse IgG-HRP and anti-rabbit IgG-HRP (1:10.000 Santa Cruz Biotechnology Inc) were used as secondary antibodies.

### Chemiluminescent immunometric assay

After 24 hs of culture, supernatants from 4μ8c- and GSK 2606414-pretreated or not KSHV-infected and mock control macrophages were collected and Interleukin-10 (IL-10), vascular endothelial growth factor (VEGF), Interleukin-8 (IL-8), Interleukin-6 (IL-6) and Interferon gamma (IFN-γ) were measured by Magnetic Luminex assay performed by R&D systems a Bio-Techne brand, using a human pre-mixed multi-analyte kit (R&D systems Bio-Techne, LXSAHM) according to the manufacturer’s instructions.

### Densitometric analysis

The quantification of proteins bands was performed by densitometric analysis using the Image J software, which was downloaded from NIH web site (http://imagej.nih.gov).

### Statistical analysis

Data are represented by the mean ± standard deviation (SD) of at least three independent experiments and two-tailed Student’s *t*-test was used for statistical significance of the differences between treatment groups. Difference was considered statistically significant when *p*-value was ≤ 0.05.

## Results

### KSHV infection reduces the survival of macrophages and skews their phenotype towards M2- like/TAM profile

**Fig. 1:**
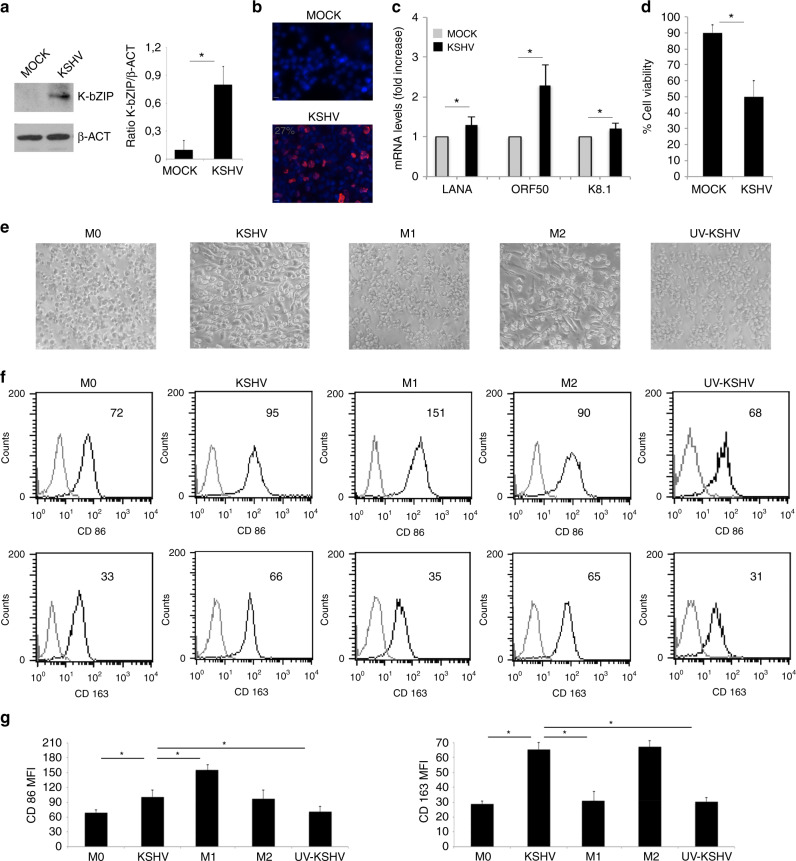
KSHV infects macrophages and skews their phenotype towards an M2 phenotype. **a** K-bZIP expression in KSHV- and mock-infected macrophages was evaluated after 24 h of infection by western blot and **b** the percentage of K-bZIP expressing cells was assessed by IFA. β-actin (β-ACT) was used as loading control. A representative experiment out of three is shown. Histograms represent the mean plus S.D. of the densitometric analysis of the ratio of K-bZIP/β-actin; **c** LANA, ORF50 and K8.1 mRNA were evaluated by qRT-PCR. The amount of target mRNA was normalised towards the β-actin gene and analysed by comparing mock and KSHV-infected macrophages. Data are plotted in histograms and standard deviation (SD)is also reported. *p-value < 0.05. **d** Cell viability of mock or KSHV-infected macrophages was studied by trypan blue exclusion assay. Mean plus SD of three independent experiments is reported. *p-value < 0.05; **e** Morphology of M0, KSHV-infected, M1, M2 and UV-KSHV-treated macrophages was observed utilising an optical microscope (×40 magnification); **f** FACS analysis of CD86 and CD163 expression of M0, KSHV-infected, M1, M2 and UV-KSHV-treated macrophages. A representative experiment is shown, and the mean of fluorescence intensity is indicated. Grey peaks represent the isotype controls. **g** Histograms representing the mean plus SD of CD86 and CD163 MFI (Mean fluorescence Intensity) are also reported. *p-value < 0.05.

**Fig. 2:**
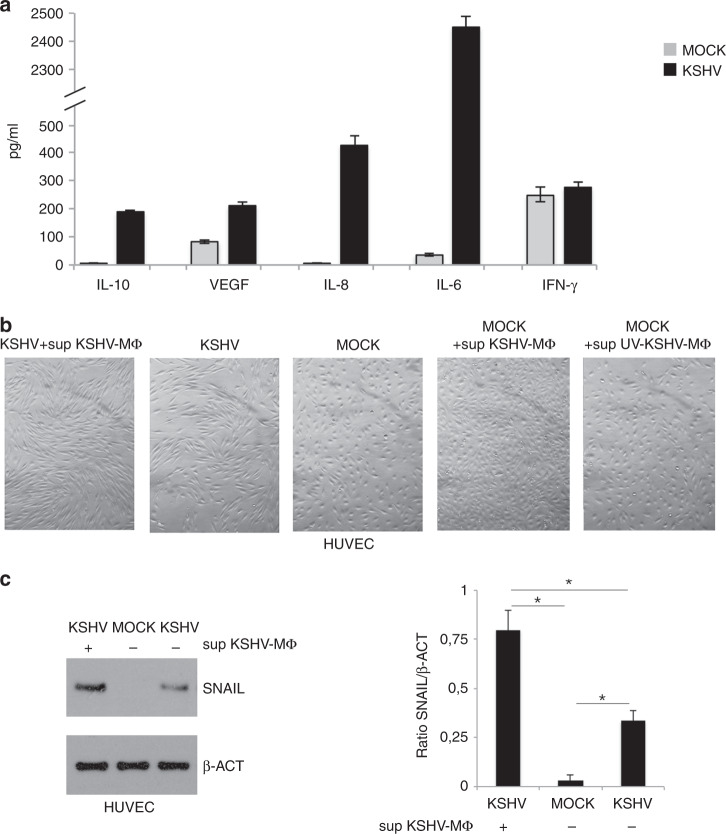
KSHV infection promotes the release of pro-tumorigenic cytokines. **a** IL-10, VEGF, IL-8, IL-6 and IFN-γ released by KSHV- and mock-infected macrophages were measured by Luminex assay. Mean plus SD of three different experiments is reported. *p-value < 0.05; **b** Morphology of KSHV-infected HUVEC plus supernatant of KSHV-infected macrophages (sup KSHV-MΦ), KSHV-infected HUVEC, Mock HUVEC, Mock HUVEC treated with supernatant of KSHV-infected macrophages (sup KSHV-MΦ) or treated with supernatant of UV-KSHV-exposed macrophages (sup UV-KSHV-MΦ) was observed utilising an optical microscope (×40 magnification); **c** Snail expression in mock-, KSHV-infected and KSHV-infected HUVEC plus sup KSHV-MΦ was evaluated by western blot analysis. β-actin (β-ACT) was used as loading control. A representative experiment out of three is shown. Histograms represent the mean plus S.D. of the densitometric analysis of the ratio of snail/β-actin. *p-value < 0.05.

Human primary CD14-positive monocytes, isolated from healthy donors and differentiated into macrophages after 6 days of exposure to M-CSF, were infected by KSHV, as previously described.^3^ After 24 h, viral infection was demonstrated by detecting the expression of the KSHV early lytic antigen K-bZIP by western blot (Fig. 1a) and by IFA (Fig. 1b) and by qRT-PCR assessing the expression of latent, early lytic and late lytic antigens, LANA, ORF50 and K8.1, respectively (Fig. 1c). We then observed that viral infection reduced the survival of macrophages (Fig. 1d) and investigated whether it could also affect their polarisation, as macrophages are in a dynamic state of activation that ranges from the classically activated M1 to a group of alternatively activated cells called M2.^8^ As suggested by cell morphology (Fig. 1e) and by the expression of surface markers (Figs. 1f, g), KSHV skewed macrophages polarisation towards M2-like/TAM profile. Indeed these cells similarly to M2 displayed a more elongated phenotype^30^ and up-regulated the expression of CD163, while slightly affected CD86 expression. CD86 and CD163 are molecules expressed mainly by M1 macrophages treated with LPS/IFN gamma or by M2 macrophages exposed to IL-4, respectively. UV-inactivated KSHV was not able to alter macrophage phenotype, suggesting that infection was required to induce the above-described effects (Fig. 1e–g). One of the most important features through which macrophages shape immune response is through the release of cytokines, therefore we next evaluated their production by mock- and KSHV-infected macrophages. As shown in Fig. 2a, viral infection increased the released of pro-tumorigenic cytokines including the immune suppressive IL-10 and VEGF and the pro-inflammatory cytokines IL-6 and IL-8. Interestingly, VEGF and IL-8 are known to exert also a strong pro-angiogenetic effect^31,32^ and contribute to the pathogenesis of KS.^33,34^ On the other hand, we found that KSHV did not increase the production of IFN-γ, cytokine that when released by macrophages^35^ can act in an autocrine fashion and stimulate their killing activity.^36^ These results suggest that the KSHV infection altered the pattern of cytokine secretion by macrophages, increasing the release of immune suppressive and pro-tumorigenic cytokines. To verify that the cytokines released by infected macrophages could effectively promote viral-driven tumorigenesis, we evaluated the effect of supernatants derived from infected macrophages in the process of KSHV-mediated transformation of HUVEC cells into spindle cells that resemble Kaposi’s Sarcoma cells.^37^ We found that HUVEC acquired a stronger spindle-like phenotype in the presence of supernatant of KSHV-infected macrophages (Fig. 2b) and expressed higher level of snail, a typical endothelial to mesenchymal transition marker (Fig. 2c), whose expression is known to increase in these cells following KSHV-infection.^38^ When the supernatants from KSHV-infected macrophages or from UV-virus-exposed macrophages were used to treat uninfected HUVEC, we found that their morphology was only slightly affected (Fig. 2b), suggesting that the factors released by KSHV-infected macrophages were able to contribute to the virus-induced transformation in spindle cells rather than alter per se the HUVEC phenotype.

### KSHV infection activates STAT3 and to a lesser extent STAT6 in macrophages while slightly affects STAT1 phosphorylation

**Fig. 3:**
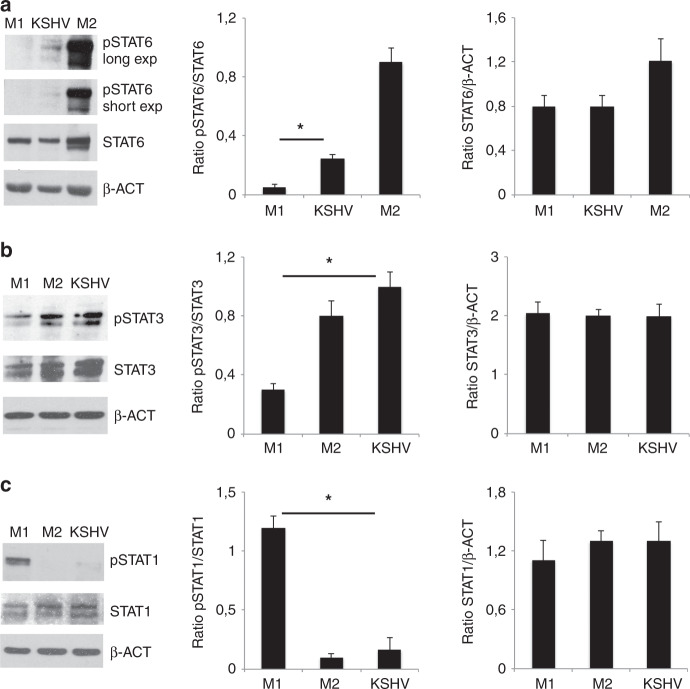
KSHV infection activates STAT3 and STAT6 in macrophages. **a** STAT6, **b** STAT3 and **c** STAT1 activation in M1, M2 and KSHV-infected macrophages was evaluated by western blot analysis. β-actin (β-ACT) was used as loading control. A representative experiment out of three is shown. Histograms represent the mean plus S.D. of the densitometric analysis of the ratio of pSTAT/STAT and STAT/ β-ACT. For pSTAT6 the short exposure was used for densitometric analysis. *p-value < 0.05 was calculated between KSHV-infected macrophages and M1 polarised cells.

We investigated whether KSHV-induced M2-like/TAM polarisation could correlate with the activation of the molecular pathways usually activated in M2-like/TAM cells. We found that viral infection increased the phosphorylation of STAT3 and to a lesser extent of STAT6, molecules activated in M2 polarised macrophages (Fig. 3a, b). Conversely STAT1, phosphorylated in M1-polarised macrophages exposed to LPS plus IFN-γ, was almost unaffected by KSHV-infection (Fig. 3c). These results indicate that the virus was able to phosphorylate somehow the molecular pathways typically activated in M2-polarised macrophages.

### KSHV activates UPR and up-regulates PD-L1 on the surface of infected macrophages

**Fig. 4:**
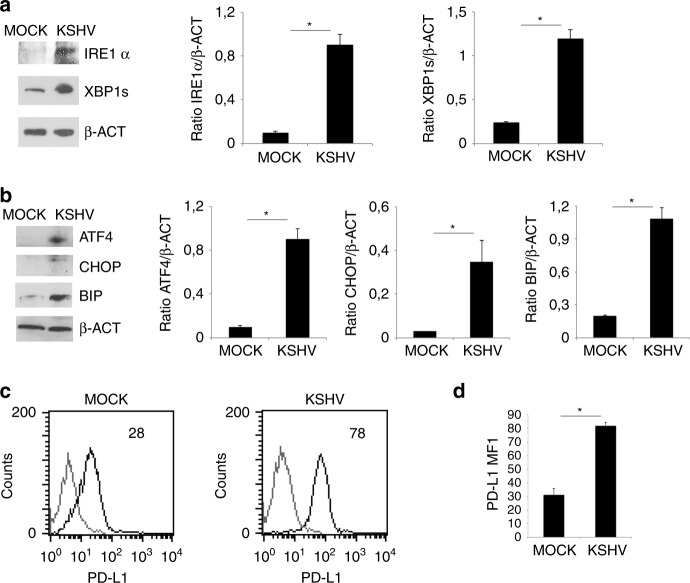
KSHV activates UPR and up-regulates PD-L1 on KSHV- infected macrophages. **a** Ire1α and XBP1s expression in mock- and KSHV-infected macrophages was evaluated by western blot analysis; **b** ATF4, CHOP and BIP expression in mock- and KSHV-infected macrophages was evaluated by western blot analysis. β-actin (β-ACT) was used as loading control. A representative experiment out of three is shown. Histograms represent the mean plus S.D. of the densitometric analysis of the ratio of each protein/β-ACT. *p-value < 0.05. **c** PD-L1 expression on mock- and KSHV-infected macrophages was evaluated by FACS analysis. A representative experiment is shown, and the mean of fluorescence intensity is indicated. Grey peaks represent the isotype controls. **d** Histograms representing the mean plus SD of PD-L1 MFI (Mean fluorescence Intensity) are also reported. *p-value < 0.05.

STAT3 and STAT6 have been reported to activate Ire1α arm of UPR and viral infection may trigger UPR by inducing ER stress or even by engaging the PRRs.^18^ Therefore, we next investigated whether KSHV could activate UPR in infected macrophages and found that the expression of Ire1α and its target XBP1s as well as ATF4 increased, suggesting that Ire1α and PERK branches of UPR were activated. In correlation with the activation of these sensors, an up-regulation of BIP and CHOP, the pro-survival and pro-death molecules of UPR, was also observed (Fig. 4a, b). As UPR triggering has been reported to up-regulate the expression of the immune checkpoint inhibitor PD-L1,^24^ we next evaluated whether KSHV could do so in infected macrophages. As shown in Fig. 4c, d, FACS analysis indicated that the expression of PD-L1 increased in KSHV-infected cells in comparison to mock-infected control, effect that may strongly contribute to the impairment of immune response.

### The inhibition of Ire1α endoribonuclease activity counteracts the release of pro-tumorigenic cytokines and PD-L1 up-regulation induced by KSHV-infection

**Fig. 5:**
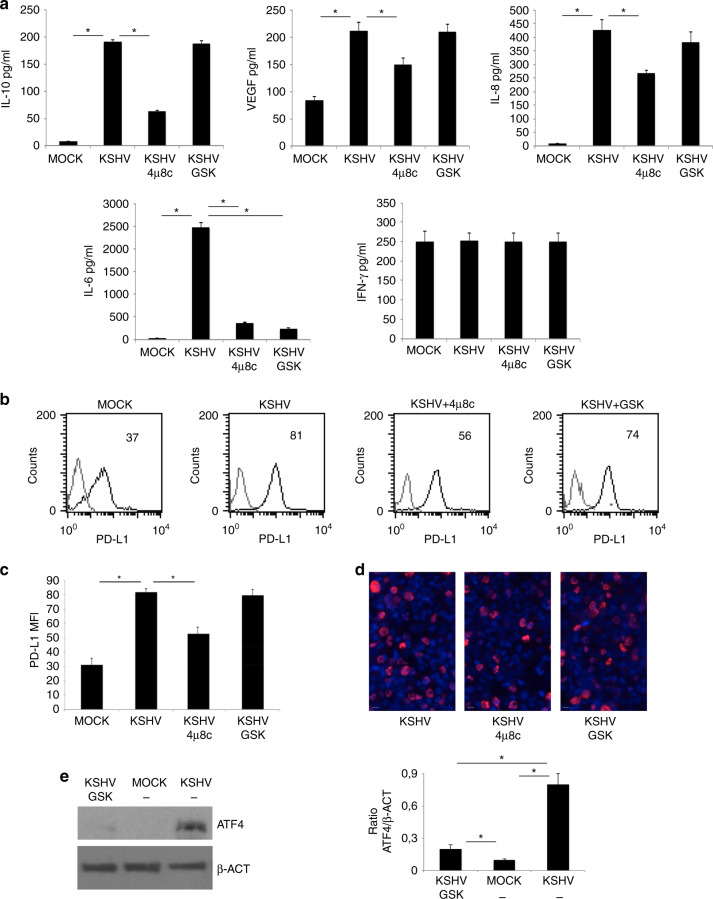
The Ire1α/XBP1 axis interferes with the pro-tumorigenic cytokines release and PD-L1 expression of KSHV-infected macrophages. **a** IL-10, VEGF, IL-8, IL-6 and IFN-γ released by mock-, KSHV-infected macrophages and 4μ8c- (Ire1α inhibitor) or GSK 2606414 (GSK)- (PERK inhibitor) pre-treated KSHV-infected macrophages were measured by Luminex assay. Mean plus SD of three different experiments is reported. *p-value < 0.05; **b** and **c** PD-L1 expression on mock-, KSHV-infected macrophages and 4μ8c or GSK 2606414 (GSK)- pre-treated KSHV-infected macrophages was evaluated by FACS analysis. Grey peaks represent the isotype controls. Histograms representing the mean plus SD of PD-L1 MFI (Mean fluorescence Intensity) are reported. *p-value < 0.05 and a representative experiment is shown, and the mean of fluorescence intensity is indicated; **d** expression of K-bZIP in untreated or 4μ8c or GSK 2606414 (GSK)- pre-treated KSHV-infected macrophages; **e** ATF4 expression in mock-, KSHV-infected and GSK 2606414 (GSK)-pre-treated KSHV-infected macrophages was evaluated by western blot analysis. β-actin (β-ACT) was used as loading control. A representative experiment out of three is shown. Histograms represent the mean plus S.D. of the densitometric analysis of the ratio of each protein/ β-ACT. *p-value < 0.05.

UPR activation may influence the cytokine release by macrophages,^18^ therefore here we next investigated the role of Ire1α and PERK activation on the production of cytokines by KSHV-infected macrophages. At this aim, we pre-treated macrophages with 4μ8C Ire1α endoribonuclease inhibitor or with GSK 2606414 (GSK) PERK inhibitor before viral infection. We observed that the release of either the immune suppressive IL-10 and VEGF and the pro-inflammatory cytokines IL-6 and IL-8 was reduced by 4μ8C while GSK only reduced IL-6 production (Fig. 5a). Finally, we found that PD-L1 surface expression was reduced by 4μ8C pre-treatment while it was slightly influenced by GSK (Fig. 5b, e). These results suggest that Ire1 α-XBP1 axis targeting could be preferentially exploited to counteract several aspects of KSHV-driven immune dysfunction and tumorigenesis. We finally assessed that such inhibitors did not interfere with KSHV infection (Fig. 5d) and evaluated whether GSK, although not able to counteract PD-L1 expression, could efficiently reduce ATF4 expression that mainly occurs downstream of PERK activation (Fig. 5e).

## Discussion

In this study, we show for the first time that KSHV infection phosphorylated STAT3 and to a lesser extent STAT6 pathways in human primary macrophages and skewed their polarisation towards M2-like/TAM profile. According to previous findings showing that STAT3 and STAT6 transcription factors activated the Ire1 α-XBP1 axis,^16^ here we found that the expression of spliced XBP1 (XBP1s) increased following KSHV infection. However, ATF4, CHOP and BIP were also up-regulated, suggesting that a more general ER stress/UPR was activated by viral infection in macrophages. Previous studies have indicated that UPR could regulate the function of immune myeloid cells such DCs and macrophages.^16,20–22^ It has been also reported that elevated UPR activation promoted M2-like polarisation of macrophages and foam cell formation^39^ or that the treatment with LPS, that induced M1 polarisation, could reduce GRP78/BIP and PERK expression.^40^ Furthermore, BIP up-regulation has been correlated with fatty acid oxidation^41^ that may occur in M2 polarised macrophages.^42^ In the last years, attention has been focused on the impact of UPR on the cytokine release by immune cells, as UPR may influence their production by activating PRR signalling or the cytokine transcription factors.^18^ Interestingly in this study, we found that Ire1 α-XBP1 axis activation by KSHV in M2/TAM polarised macrophages promoted the release of immunosuppressive as well as pro-inflammatory cytokines, while PERK activation was involved only in IL-6 production. KSKV infection of macrophages indeed increased the release of cytokines such as VEGF and IL-8 that, besides inducing immune suppression, promote angiogenesis and may sustain tumour growth.^33,34^ These cytokines, together with IL-10 and IL-6, whose release also increased following KSHV infection, play an essential role in the pathogenesis of virus-associated malignancies.^43,44^ Thus, it is possible that macrophages could be infected also in vivo by KSHV, especially within the tumour microenvironment, and the infected cells could then strongly contribute to the onset and survival of virus-associated malignancies. In support of this hypothesis, here we showed that the supernatants of virus-infected macrophages promoted KSHV-driven transformation of HUVEC into spindle cells. This result, together with previous findings showing that KSHV-infected HUVEC release factors that promoted macrophages polarisation into TAM, suggests that an active cross-talk between viral-infected HUVEC and viral-infected macrophages may occur in vivo, within the tumour bed of KS lesions. Indeed, most of the cytokines released by KSHV-infected macrophages can act in an autocrine and paracrine fashion and activate both in immune and tumour cells transcription factors such as STAT3 that plays a dual role, impairing the function of immune cells and concomitantly sustaining the growth of tumour cells.^45,46^ Of note, the interplay between STAT3 and Ire1α has been previously reported as STAT3, together with STAT6, may activate Ire1α endoribonuclease activity^16^ and Ire1α may in turn contribute to STAT3 phosphorylation.^47^ Among the numerous targets of STAT3 there is CD163,^13^ whose expression has been reported to increase in macrophages undergoing ER stress,^39^ and PD-L1,^48^ molecules found to be up-regulated on the surface of KSHV-infected macrophages. The increase of PD-L1 that may strongly contribute to viral-induced immune suppression,^49^ has been previously reported to be up-regulated by KSHV^25^ as well as by EBV infection in monocytes,^48^ suggesting that such effect may represent a common strategy used by gammaherpesvirus to impair T cell function.

In conclusions, in this study, we identified that UPR activation, particularly the Ire1α arm, was a key mechanism leading to PD-L1 up-regulation and to the release of pro-tumorigenic cytokines induced by KSHV in M2 like/TAM polarised macrophages. UPR manipulation could thus allow to re-shape infected-macrophages into a less pro-tumorigenic profile, considering that plasticity is an intriguing characteristic of these cells that are able to shift back and forth between the M1 and M2/TAM extremes, depending on the different environmental conditions to which they are subjected.^50^ This study suggests that UPR manipulation could be a promising approach to counteract the pro-tumorigenic activity of M2/TAM macrophages, besides improving the outcome of several cytotoxic anti-cancer treatments.

## Data Availability

The datasets generated and/or analysed during the current study are available from the corresponding author upon reasonable request.
